# 3D-printed graphene/polymer structures for electron-tunneling based devices

**DOI:** 10.1038/s41598-020-68288-5

**Published:** 2020-07-09

**Authors:** Deisy C. Carvalho Fernandes, Dylan Lynch, Vikas Berry

**Affiliations:** Department of Chemical Engineering, University of Illinois at Chicago, Chicago, IL USA

**Keywords:** Electronic devices, Electronic devices

## Abstract

Designing 3D printed micro-architectures using electronic materials with well-understood electronic transport within such structures will potentially lead to accessible device fabrication for ‘on-demand’ applications. Here we show controlled nozzle-extrusion based 3D printing of a commercially available nano-composite of graphene/polylactic acid, enabling the fabrication of a tensile gauge functioning via the readjustment of the electron-tunneling barrier width between conductive graphene-centers. The electronic transport in the graphene/polymer 3D printed structure exhibited the Fowler Nordheim mechanism with a tunneling width of 0.79–0.95 nm and graphene centers having a carrier concentration of 2.66 × 10^12^/cm^2^. Furthermore, a mechanical strain that increases the electron-tunneling width between graphene nanostructures (~ 38 nm) by only 0.19 Ǻ reduces the electron flux by 1e/s/nm^2^ (from 18.51 to 19.51 e/s/nm^2^) through the polylactic acid junctions in the 3D-printed heterostructure. This corresponds to a sensitivity of 2.59 Ω/Ω%, which compares well with other tensile gauges. We envision that the proposed electron-tunneling model for conductive 3D-printed structures with thermal expansion and external strain will lead to an evolution in the design of next-generation of ‘on-demand’ printed electronic and electromechanical devices.

## Introduction

Graphene—a single-atom-thick sheet of carbon atoms arranged in a hexagonal honeycomb lattice^1^—has gained considerable attention due to its exceptional mechanical^2^, electrical^3^, and thermal properties^4^. Compounding on these properties, graphene’s chemical and physical stability has led to numerous applications, including nanoelectronics, biomedical applications such as biosensors, antibacterial, drug delivery, cell imaging, tissue engineering, and energy storage applications in batteries^5,6^. Graphene and its derivatives include graphene oxide (oxygenated graphene), graphane (hydrogenated graphene), and fluorographene (fluorinated graphene). Graphene oxide is one of the most widely studied chemical derivatives of graphene because of its water solubility and fast, scalable synthesis. A fast-evolving research-field is the 2D and 3D printing of nanomaterials, including graphene oxide and its composites. 3D printing, which became an important research area in the 1980s, can significantly improve fabrication of microelectronic devices and other constructs for various fields due to the ability of on-demand manufacturing, at reduced cost in comparison to other fabrication techniques.

Further, the applications of 3D printing has evolved into the fields of biomedicine^7,8^, and sensors^9–13^. There is also an increasing number of applications in electrochemical systems such as supercapacitors, electrodes, electrochemical, batteries, and water splitting using 3D printing^14^. The Black Magic filament (used in this work) has been previously used for photoelectrochemical sensors, supercapacitor, and electrodes^15–17^. This PLA based filament has 8 wt% of graphene^18^. In this work, we show that electronic devices with graphene-composite can be 3D printed for device evaluation and strain gauge application. These electronic devices constructs can potentially have applications in wearable electronics where flexibility is essential^19–21^.

Here, we outline the electron-transport properties (transport-barrier, average tunneling distance, carrier concentration, graphene nanostructures size, electron flux) of 3D printed devices made from graphene/PLA filaments, and the effect of temperature and strain on these properties.

## Results

**Figure 1:**
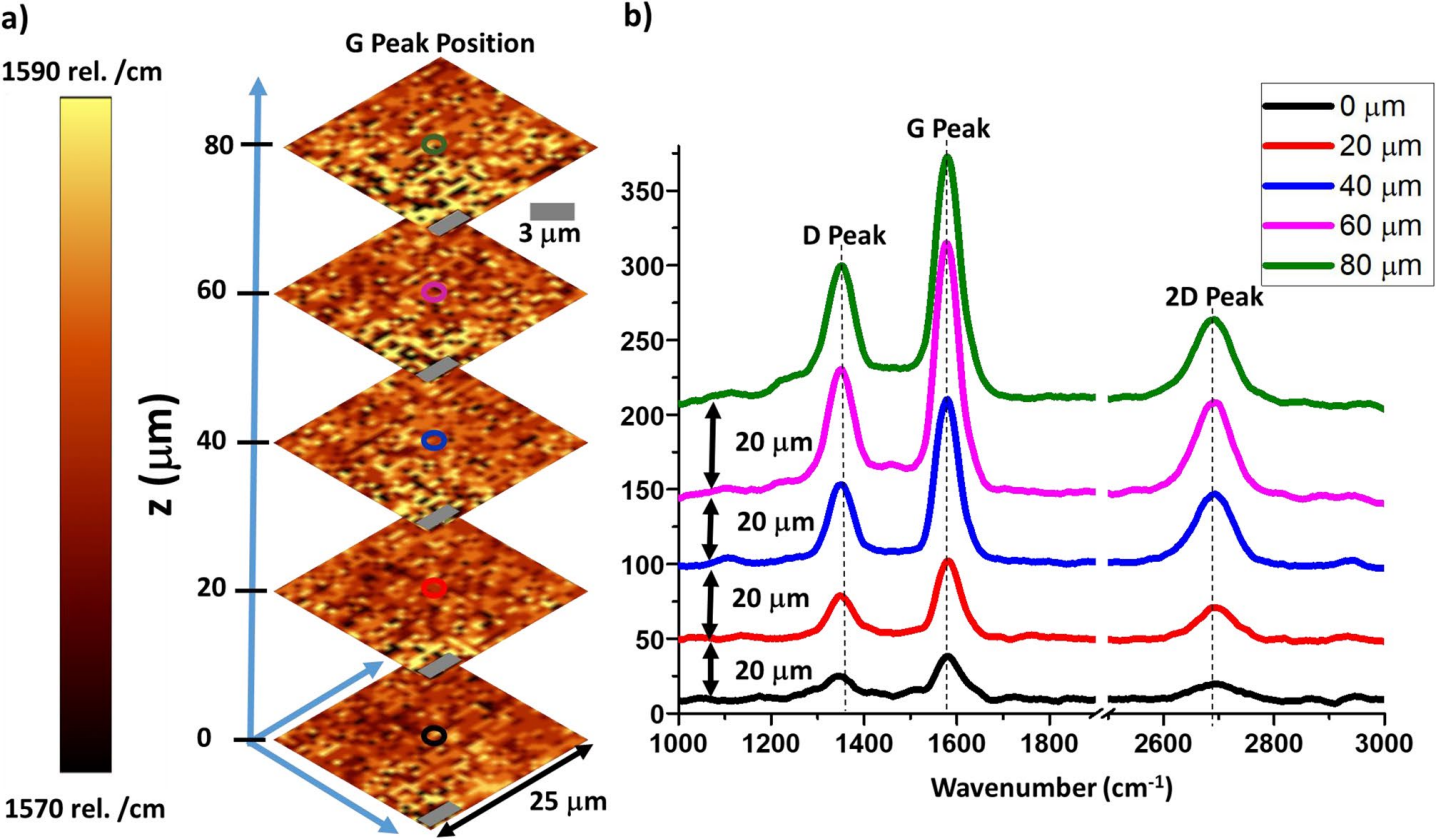
(**a**) Raman position mapping of graphene G peak at different depths of the mixed structure with each consecutively scan 20 m apart showing the consistency of the graphene present throughout the structure (**b**) Raman spectra of the stacked structure showing D, G and 2D peaks at different depths (each scan is 20 m apart) of the PLA/Graphene 3D printed structure that corresponds to the mapping showing in (**a**), indicating that the graphene sheets are preserved the same throughout the device.

Figure 1 shows the depth profiling of device 1 where the G-peak position spatial mapping and the spectra were obtained at different heights, and we observed: (a) the intensity of Raman signal from the photodetector reduces as we scan deeper into the composite (the focal plane it moved into the composite from the surface), (b) there is no relatively change with depth to the position of the D, G and 2D (1,341 ± 3 cm^−1^, 1,580 ± 1 cm^−1^, and 2,692 ± 1 cm^−1^) bands, and (c) the arrangement of the graphene platelets is random (see supplementary information); however, its interface with PLA is consistent. The decrease in Raman signal intensity with depth (Fig. 1) is attributed to the absorption of the scattered light by the material that the light passes through before reaching the objective lens.

It is known that different amounts of polymer dopes graphene differently to change its Raman peak positions^22,23^. The Raman G-peak position (scale: 1,570 to 1585 cm^−1^) is maintained at different depths in the printed graphene/PLA structure, (even at 80 m deep) (Fig. 1); this implies that the relative concentration of graphene with respect to interfaced-PLA remains nominally unchanged. Further, since the composite is conductive (further explained in the next section), the graphene-network is percolating, implying that the microscale composition of graphene is also uniform. The Raman mapping also shows that the arrangement of graphene sheets at every depth-section is random with similar coverage of graphene (Fig. 1 and supplementary figure). Importantly, the printing process does not modify the dispersion of graphene within the PLA matrix.

When the measurements were made at different depths, we confirmed that graphene exists at every depth. However, since graphene is a large nanomaterial (several microns in lateral size), its presence in a composite cannot be uniform in the micron scale. We found that its composition is uniform in the 50 × 50 micron^2^ area-scale.

Using the Tuinstra and Koenig^24^ relationship, the graphitic size of the samples were calculated using the I_D_/I_G_ intensity ratio:

1 $$ \frac{I\left( D \right)}{{I\left( G \right)}} = \frac{{C^{\prime } \left( \lambda \right)}}{{L_{a} }} $$

where *L*_*a*_ is the in-plane correlation length or cluster diameter, $C^{\prime } \left( \lambda \right)$ is the variable scaling coefficient, *I*(*D*)*,* and *I*(*G*) are the intensity of D and G peak, respectively. $C^{\prime } \left( \lambda \right)\sim 19.22$ nm was calculated according to Cançadoa et al.^25^, I(D), and I(G) was obtained from the Raman graph (from the integration of the area under the curve), indicating that the ordered graphitic region with sp^2^ hybridized carbon atoms in the graphene sheets is in the order of 37.75 ± 2.42 nm (*L*_*a*_). In addition, using the relationship^26^:

2 $$ \overline{h} \Delta Pos \left( G \right)_{{E_{F} }} = \frac{{\lambda_{{\Gamma }} }}{2*\pi }\left[ {\left| {E_{F} } \right| + \frac{{\overline{h}Pos\left( G \right)_{0} }}{4} ln\left| {\frac{{E_{F} - \overline{h}Pos\left( G \right)_{0} }}{{E_{F} + \overline{h}Pos\left( G \right)_{0} }}} \right|} \right] $$

as $\overline{h}$ is the Planck’s constant, *Pos(G)* is the position of the G peak (derived from the Lorentz fitting), *Pos*(*G*)_0_ is the position of the G peak without doping, $\lambda_{{\Gamma }}$ is the dimensionless electron–phonon coupling for the LO phonons at ${\Gamma }$ (the value used was 0.03^26^), and $E_{F}$ is the Fermi energy distribution of doping. It is important to note that several different values of *Pos*(*G*)_0_ have been reported in the literature (See Supporting Information) with an average value of 1583.67 cm^−1^^27–35^. Using Eq. 2 and the values of the G peak positions for the different depths, we found the different values of E_F_ (0.21 eV). The doping concentration was calculated using^36^:

3 $$ \left| {E_{F} \left( n \right)} \right| = \overline{h}\left| {v_{F} } \right|\sqrt {\pi n} $$

where $v_{F}$ is fermi level velocity (1.1 × 10^6^ m/s), and $n$ is the carrier concentration. Equations 2 and 3 were solved in Matlab using the values from the Raman spectra, obtaining the value of the carrier concentration average of 2.63*10^12^/cm^2^. This order of magnitude of doping is consistent with that of other graphenic composites. Equations 2 and 3 are derived for graphene with large number of sp^2^ hybridization of carbon atoms (infinite sp^2^ carbon lattice); however, the relatively sp^2^ domain size of graphene in this study is ~ 37.75 nm (or ~ 54,000 sp^2^ carbon atoms per domain). Therefore, the validity of these equations and the derived charge density is limited.

**Figure 2:**
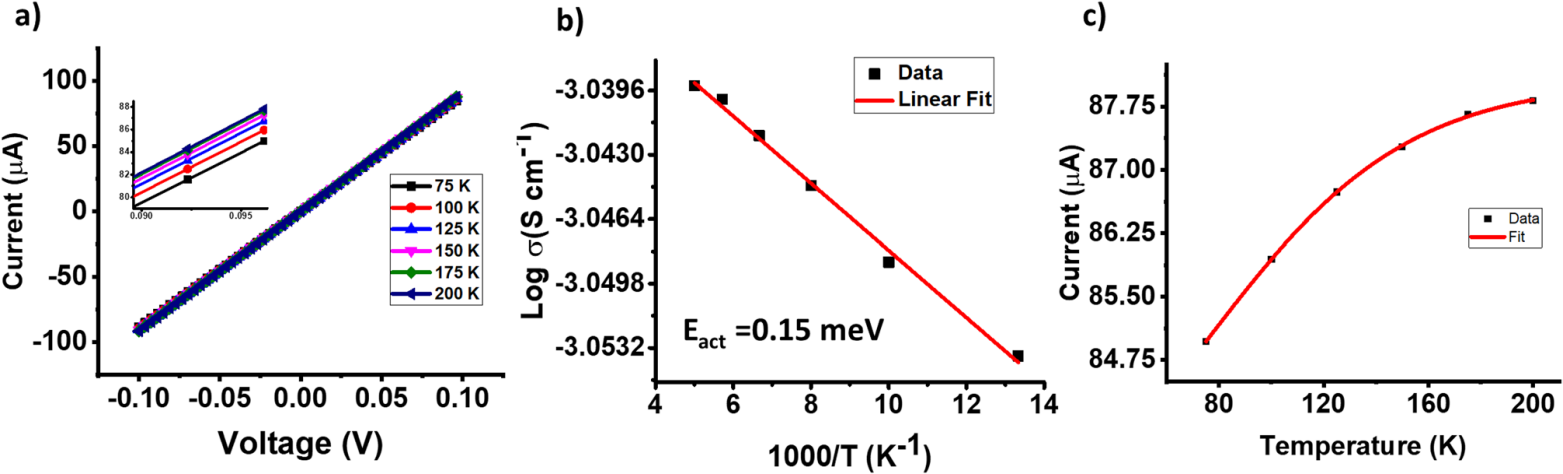
(**a**) Current–voltage characteristic of the mixed graphene/PLA structure (device1) at different temperatures ranging from 75 to 200 K, (**b**) Arrhenius plot of the conductivity obtained from the data in figure (**a**) giving activation energy of 0.15 meV. (**c**) Current–temperature plot showing the Fowler Nordheim conduction fit with a tunneling distance of 0.78 nm at 0 K.

The electron transport for the percolating network of the 3D printed structure of device 1 was studied using a cryo-probe-station under vacuum (0.75 mTorr), acquiring the current–voltage (*I*–*V*) data of device 1 at different temperatures. Figure 2 shows the I–V characteristic of the device measured at 75 K, 100 K, 125 K, 150 K, 175 K, and 200 K.

To obtain the overall transport thermal-barrier of the 3D printed devices for electron transfer between graphene platelets, we applied Arrhenius Law to fit the I–V data. This was done to determine the mechanism that most appropriately describes the electron transport (electron tunneling or thermal hopping) (shown in Supplementary v4Information) :

4 $$ \left( {\frac{I}{V} \upmu {\text{ exp}}\left( { - \frac{{E_{a} }}{{K_{B} T}}} \right)} \right) $$

where *Ea* is the thermal barrier height, *k*_*B*_ is the Boltzmann constant, and *T* is the temperature (details of this calculation are shown in supplementary information). Using the I–V (Fig. 2a) data obtained at different temperatures under high vacuum (0.75 mTorr) and fitting the impedance with the Arrhenius equation (Fig. 2b) we found the thermal barrier height of 0.15 meV. This is smaller than *k*_*B*_*T* at room temperature (25 meV). Since thermal emission occurs with thermal barriers higher than *k*_*B*_*T* at room temperature, the mechanism of carrier transportation for this device is electron tunneling^37,38^. Electron-tunneling is a phenomena that occurs when the electron potential is below the barrier height and therefore, can occur at low-electrical fields (or low electron potentials) as shown by Nakatsuji et al.^39^, Takayanagi et. al.^40^, Wang et. al.^41^, and Nakatsuji et al.^42^.

For electron-tunneling within this thermally-expandable network, we included a thermal-expansion equation with the Fowler Nordheim electron tunneling (FNET) Eq. ^37,38,43–46^. Consistently, Connelly et al. also employed the Fowler–Nordheim Model for electron transport within their 3D printed structure^47^.

In this work, the thermal expansion equation applied on the interparticle polymer layers governs the tunneling distance:

5 $$ \frac{a}{{a_{0} }} = 1 + {\text{a}} \times {\text{T}} $$

where *a*_0_ and *a* are the average tunneling distance at zero-Temperature and at any other temperature, and is the thermal expansion coefficient.^48^ Combing with the FNET equation with Eq. 5, we get

6 $$ I = I_{0} + t {\exp}\left( {\left( {\frac{{2\left( {2m\emptyset } \right)^{\frac{1}{2}} }}{{\overline{h}}}} \right)a_{0} *\left( {1 + {\text{a}} \times {\text{T}}} \right)} \right) $$

where *m* is the mass of an electron, *t* is the FNET constant, and is the tunneling barrier height. Since the electron must dissociate from graphene before tunneling into the next graphene platelet, the tunneling barrier is assumed to be graphene’s work function: 4.85 eV. Fitting the data, as shown in Fig. 2c, the tunneling distance at absolute zero temperature was found to be 0.78 nm (calculation-details are provided in Supplementary Information).

**Figure 3:**
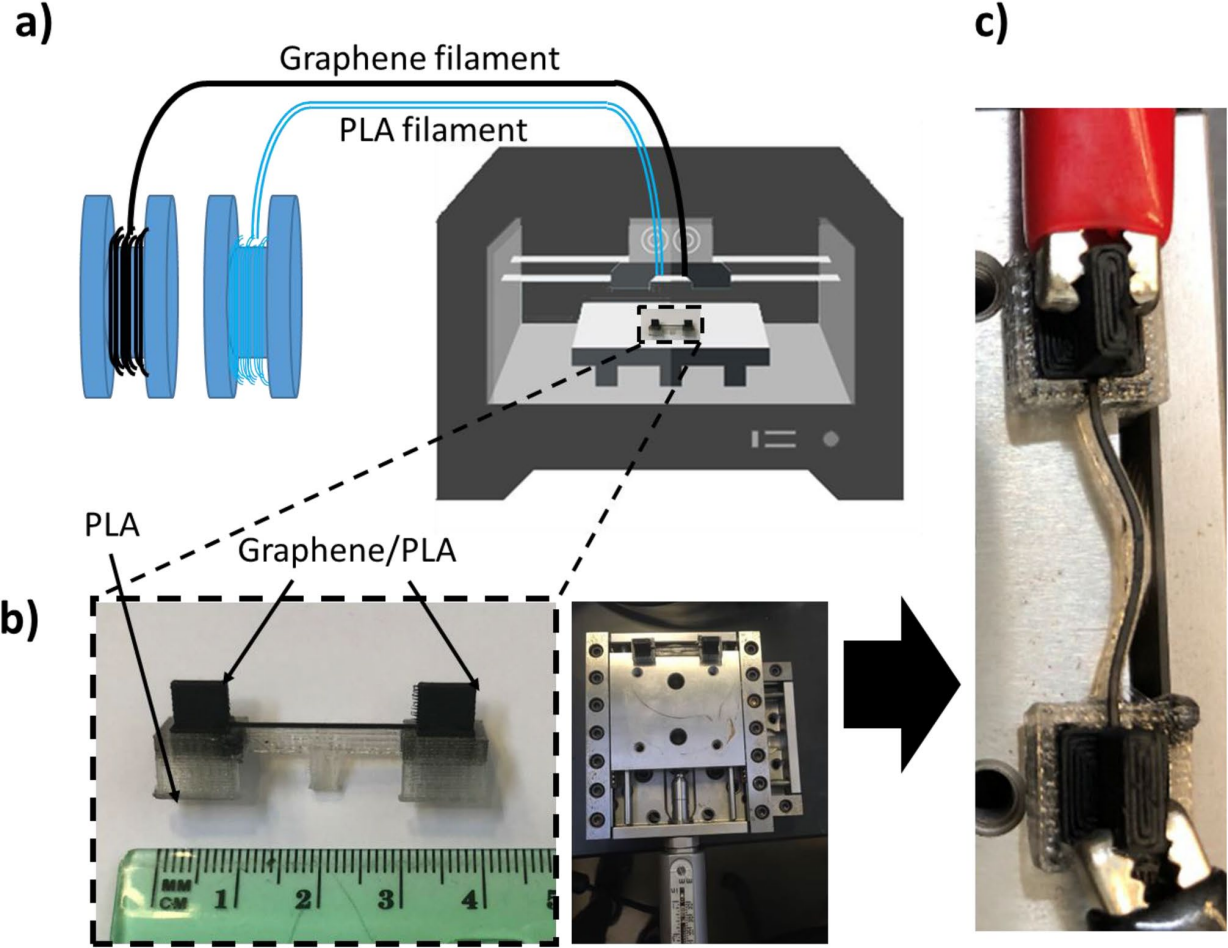
(**a**) Schematic of the 3D printer process of the graphene/PLA structure for strain test, (**b**) a picture of the 3D printed device 2 used for strain testing, (**c**) image of strained device 2 with electrodes connected to the 3D printed structure.

The strain was induced on device 2 (the channel length, width, and height are 8 mm, 0.8 mm, and 0.4 mm respectively, and the electrode dimensions are 2 × 3 × 6 mm^3^) by a motion controller driver and the strain analysis of the 3D printed graphene/PLA structure was performed. Here, the device was fixed on a lever with the motion-controller driver pushing on the graphene/PLA to generate strain, and two electrodes were connected to the edges of the devices, as shown in Fig. 3. The strain percentage was calculated via:

7 $$ \varepsilon = \frac{{\left( {2{\text{* sin}}^{ - 1} \left( {\frac{{{\raise0.7ex\hbox{${l_{0} }$} \!\mathord{\left/ {\vphantom {{l_{0} } 2}}\right.\kern-\nulldelimiterspace} \!\lower0.7ex\hbox{$2$}}}}{{\left( {\frac{{l_{0}^{2} }}{8h} + \frac{h}{2}} \right)}}} \right)} \right)* \left( {\frac{{l_{0}^{2} }}{8h} + \frac{h}{2}} \right) - l_{0} }}{{l_{0} }} \times 100 $$

where *ε* is the strain, $l_{0}$ is the length of the device at rest (with no strain applied), and *h* is the bending-distance after strain is applied. Details of this calculation and a figure to illustrate it is shown in the Supplementary Information.

**Figure 4:**
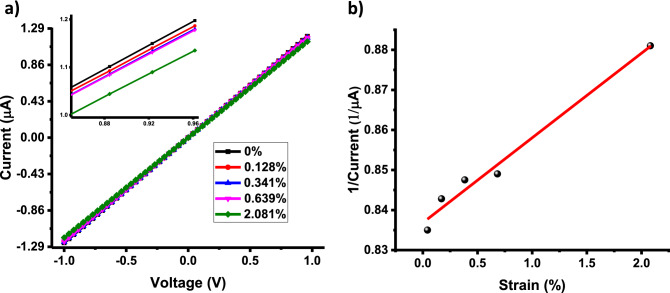
(**a**) Current–voltage relationship for the c graphene/PLA structure (device2) at room temperature varying levels of strain from 0.0 to 2.038%. (**b**) Current–strain plot and the Fowler Nordheim fit of with a tunneling distance of 0.95 nm at room temperature (voltage applied = 1 V).

The *I*–*V* measurements on the strained device were performed for different values of strain (Fig. 4). The electron-transport on this device also follows FNET, and it was combined with the strain equation to obtain:

8 $$ I = I_{0} + t\exp \left( { - \left( {\frac{{2\left( {2m\emptyset } \right)^{\frac{1}{2}} }}{{\overline{h}}}} \right)a_{0} \times \left( {1 + \frac{{\text{e}}}{100}} \right)} \right) $$

where the tunneling barrier (*ϕ*) is kept as the work function of graphene (4.85 eV). Fitting the above equation with the current versus the strain data (Fig. 4, regression = 0.994) provides the tunneling distance at rest at room temperature to be 0.95 nm (calculations shown in Supplementary Information). This indicates that the device can be used as a strain gauge. Extrapolating from the equation for electron-tunneling with thermal expansion (Eq. 6) for the tunneling distance at room temperature, we get 0.95 nm, consistent with the result from the strain equation (Eq. 8). The electron flux $(e_{f} = I/(e.A_{cross - section} ))$ on device 2 between the graphene sheets under 0% strain is 18.51 e/s/nm^2^, and the electron flux under 2.038% strain is 19.51 e/s/nm^2^; this shows that increasing the electron tunneling width between the nanostructures by just 0.19 Angstrom the electron flux reduces by 1 e/s/nm^2^ through the polylactic acid junctions in the 3D-printed heterostructure. Also, for our device, the change in resistance is expressed as $(\frac{{R_{0} - R}}{{R_{0} }})$, where $R_{0}$ is the resistance of the unstrained device (ε = 0%), R is the strained device (ε = 2.038%). This value is 5.28 ± 0.15%. This results in the response sensitivity, $(\frac{{R_{0} - R}}{{R_{0} }}){/}(\% Strain)$, of 2.59 Ω/Ω%. Other works have reported similar response sensitivity values: Tian et al.^49^ reports ~ 0.12 Ω/Ω%, Bae et. al.^50^ reports ~ 0.3 Ω/Ω%, Xu et. al.^51^ reports 1 Ω/Ω%, Wang et. al.^52^ reports ~ 3 Ω/Ω%, and Jinlong et. al.^53^ reports ~ 4 Ω/Ω%.

## Discussion

In conclusion, we demonstrate 3D printed structures of graphene/PLA, applicable as components of on-demand electronic devices. We show the operation of a tensile gauge functioning via the modification of electron-tunneling width between graphenic-centers. For the graphene/PLA system, the thermal barrier to electron transport was 150 µeV (much smaller than energy at room temperature), and the electron tunneling distance was 0.78 nm at cryo-temperature and 0.95 nm at room temperature. The mechanical strain that increases the electron-tunneling width between graphene nanostructures (~ 38 nm) by an average of 0.19 Angstrom reduces the electron flux from 18.51 to 19.51 e/s/nm^2^ for the 3D-printed heterostructure. Our work shows that a 3D printable filament with a network of 2D nanomaterials (with low percolation threshold) within the polymer matrix can be building blocks for on-demand electronic devices.

## Methods

**Figure 5:**
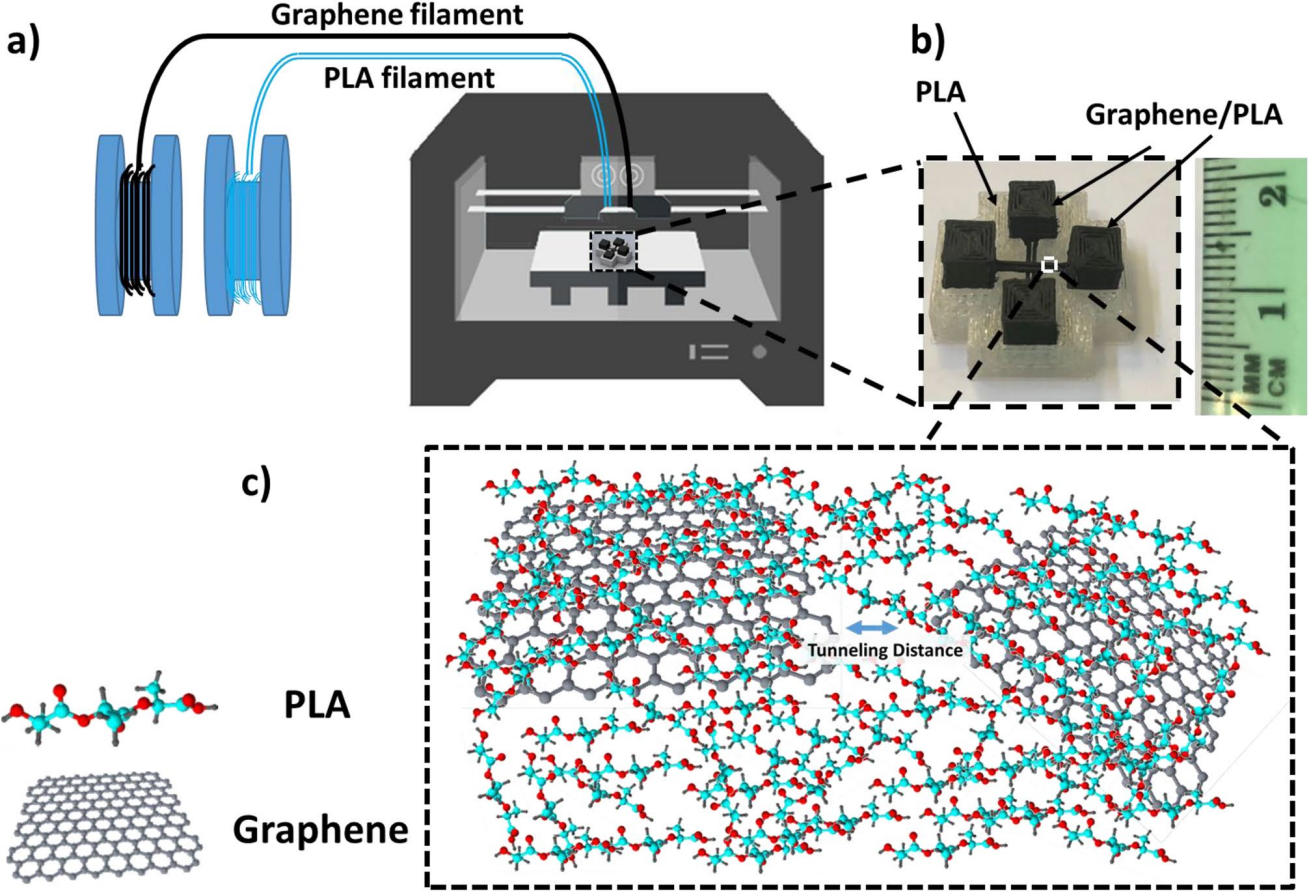
(**a**) Schematic of 3D printing process of graphene sheets/PLA for device 1 used for electrical conductivity studies (**b**) image of device 1 used for Raman spectroscopy and investigating temperature dependence of electrical conductivity, (**c**) model of the chemical structure of device 1 showing the schematic of the graphene/PLA distribution throughout the 3D printed structure.

In this report, we fabricated two different devices for investigating the effects of temperature (device 1) and mechanical strain (device 2) on the electrical conductivity of the 3D printed graphene/PLA nano-composite structures. The devices were designed in Autodesk Inventor and loaded to the CTC Bizer series Dual Nozzle 3D Printer (0.4 mm nozzle size, 1.75 mm filament size (GRPHN-PLA, Black Magic 3D), Printer setting: stage-temperature at 60 °C, and extruder temperature at 190 ° C) for device-fabrication. The schematic of the mechanism to 3D printed devices is shown in Fig. 5. The printing time for the graphene/PLA structure of device 1 was 9 min (18 min for the PLA support structure), the channel length, width, and height are 8 mm, 1 mm, and 0.4 mm respectively, and the electrode dimensions are 5 × 5 × 5 mm^3^. (Follow the same process for device 2). The structure and spatial distribution of graphene in the printed composite devices were characterized employing confocal Raman spectroscopy (WITEC Alpha-300-RA system with 532 nm incident laser and 100X objective lens). The Raman spectra of the devices also provided information on the doping levels of the graphene as well as the size of the ordered graphitic regions.

## Supplementary information

Supplementary file1 (PDF 424 kb)

## References

[1] Novoselov KS (2004). Electric field effect in atomically thin carbon films. Science.

[2] Lee C, Wei X, Kysar JW, Hone J (2008). Measurement of the elastic properties and intrinsic strength of monolayer graphene. Science.

[3] Zhang YB, Tan YW, Stormer HL, Kim P (2005). Experimental observation of the quantum Hall effect and Berry’s phase in graphene. Nature.

[4] Balandin AA (2008). Superior thermal conductivity of single-layer graphene. Nano Lett..

[5] Pattnaik S, Swain K, Lin Z (2016). Graphene and graphene-based nanocomposites: biomedical applications and biosafety. J. Mater. Chem. B.

[6] Huang X (2011). Graphene-based materials: synthesis, characterization, properties, and applications. Small.

[7] Gross BC, Erkal JL, Lockwood SY, Chen C, Spence DM (2014). Evaluation of 3D printing and its potential impact on biotechnology and the chemical sciences. Anal. Chem..

[8] Mannoor MS (2013). 3D printed bionic ears. Nano Lett..

[9] Xu Y (2017). The boom in 3D-printed sensor technology. Sensors..

[10] Rahman MT, Moser R, Zbib HM, Ramana CV, Panat R (2018). 3D printed high performance strain sensors for high temperature applications. J. Appl. Phys..

[11] Zhu C (2015). Highly compressible 3D periodic graphene aerogel microlattices. Nat. Commun..

[12] Hong-Bin Y (2013). A flexible and highly pressure-sensitive graphene–polyurethane sponge based on fractured microstructure design. Adv. Mater..

[13] Eswaraiah V, Balasubramaniam K, Ramaprabhu S (2011). Functionalized graphene reinforced thermoplastic nanocomposites as strain sensors in structural health monitoring. J. Mater. Chem..

[14] Foo CY, Lim HN, Mahdi MA, Wahid MH, Huang NM (2018). Three-dimensional printed electrode and its novel applications in electronic devices. Sci. Rep..

[15] O’Neil GD (2019). Single-step fabrication of electrochemical flow cells utilizing multi-material 3D printing. Electrochem. Commun..

[16] Grande L (2012). Graphene for energy harvesting/storage devices and printed electronics. Particuology.

[17] Browne MP, Pumera M (2019). Impurities in graphene/PLA 3D-printing filaments dramatically influence the electrochemical properties of the devices. Chem. Commun..

[18] Foster CW (2017). 3D Printed graphene based energy storage devices. Sci. Rep..

[19] Muth JT (2014). Embedded 3D printing of strain sensors within highly stretchable elastomers. Adv. Mater..

[20] Chun S (2014). A graphene force sensor with pressure-amplifying structure. Carbon N. Y..

[21] Chen S, Jiang K, Lou Z, Chen D, Shen G (2018). Recent developments in graphene-based tactile sensors and E-skins. Adv. Mater. Technol..

[22] Francis C (2017). Raman spectroscopy and microscopy of electrochemically and chemically doped high-mobility semiconducting polymers. J. Mater. Chem. C.

[23] Kalbáč M, Kavan L, Zukalová M, Dunsch L (2007). An in situ Raman spectroelectrochemical study of the controlled doping of single walled carbon nanotubes in a conducting polymer matrix. Carbon N. Y..

[24] Tuinstra F, Koenig JL (1970). Raman spectrum of graphite. J. Chem. Phys..

[25] Cançado LG (2006). General equation for the determination of the crystallite size La of nanographite by Raman spectroscopy. Appl. Phys. Lett..

[26] Ferrari AC, Basko DM (2013). Raman spectroscopy as a versatile tool for studying the properties of graphene. Nat. Nanotechnol..

[27] Li X (2009). Large-area synthesis of high-quality and uniform graphene films on copper foils. Science.

[28] Ferrari AC (2006). Raman spectrum of graphene and graphene layers. Phys. Rev. Lett..

[29] Ouyang Y, Chen L (2011). Surface-enhanced Raman scattering studies of few-layer graphene on silver substrate with 514 nm excitation. J. Mol. Struct..

[30] Fu X, Bei F, Wang X, O’Brien S, Lombardi JR (2010). Excitation profile of surface-enhanced Raman scattering in graphene–metal nanoparticle based derivatives. Nanoscale.

[31] Di C (2008). Patterned graphene as source/drain electrodes for bottom-contact organic field-effect transistors. Adv. Mater..

[32] Das A (2008). Monitoring dopants by Raman scattering in an electrochemically top-gated graphene transistor. Nat. Nanotechnol..

[33] Lee I (2014). Poly-4-vinylphenol and poly(melamine-co-formaldehyde)-based graphene passivation method for flexible, wearable and transparent electronics. Nanoscale.

[34] Lucchese MM (2010). Quantifying ion-induced defects and Raman relaxation length in graphene. Carbon N. Y..

[35] Wang H, Wang Y, Cao X, Feng M, Lan G (2009). Vibrational properties of graphene and graphene layers. J. Raman Spectrosc..

[36] Das A (2008). Monitoring dopants by Raman scattering in an electrochemically top-gated graphene transistor. Nat. Nanotechnol..

[37] Sreeprasad TS (2013). Electron-tunneling modulation in percolating network of graphene quantum dots: fabrication, phenomenological understanding, and humidity/pressure sensing applications. Nano Lett..

[38] Sreeprasad TS (2015). Graphene quantum dots interfaced with single bacterial spore for bio-electromechanical devices: a graphene cytobot. Sci. Rep..

[39] Nakatsuji H, Shimizu A, Omura Y (2000). Semi-empirical and practical model for low-electric field direct tunneling current estimation in nanometer-thick SiO2films. Superlattices Microstruct..

[40] Takayanagi M, Iwabuchi S (1991). Theory of band-to-band tunneling under nonuniform electric fields for subbreakdown leakage currents. IEEE Trans. Electron. Devices.

[41] Wang PY, Tsui BY (2016). Band engineering to improve average subthreshold swing by suppressing low electric field band-to-band tunneling with epitaxial tunnel layer tunnel FET structure. IEEE Trans. Nanotechnol..

[42] Nakatsuji H, Omura Y (1999). Practical model for low electric field direct-tunnelling current characteristics in nanometer-thick oxide films. Electron. Lett..

[43] Murphy EL, Good RH (1956). Thermionic emission, field emission, and the transition region. Phys. Rev..

[44] Schuegraf KF, Hu C (1994). Metal-oxide-semiconductor field-effect-transistor substrate current during Fowler–Nordheim tunneling stress and silicon dioxide reliability. J. Appl. Phys..

[45] Forbes RG (2006). Simple good approximations for the special elliptic functions in standard Fowler–Nordheim tunneling theory for a Schottky–Nordheim barrier. Appl. Phys. Lett..

[46] Berry V, Saraf RF (2011). Modulation of electron tunneling in a nanoparticle array by sound waves: an avenue to high-speed, high-sensitivity sensors. Small.

[47] Connelly JM, Tang WW, Harris JR, Jensen KL (2019). Demonstration of 3-D-printed field-emission cathodes. IEEE Trans. Plasma Sci..

[48] Botean, A.-I. Thermal expansion coefficient determination of polylactic acid using digital image correlation. *E3S Web Conf.* **32**, (2018).

[49] Tian H (2014). Scalable fabrication of high-performance and flexible graphene strain sensors. Nanoscale.

[50] Bae S-H (2013). Graphene-based transparent strain sensor. Carbon N. Y..

[51] Xu X (2015). Self-sensing, ultralight, and conductive 3d graphene/iron oxide aerogel elastomer deformable in a magnetic field. ACS Nano.

[52] Wang Y (2014). Wearable and highly sensitive graphene strain sensors for human motion monitoring. Adv. Funct. Mater..

[53] Jinlong L, Meng Y, Suzuki K, Miura H (2017). Fabrication of 3D graphene foam for a highly conducting electrode. Mater. Lett..

