# Supplementary material for: 3D-printed graphene/polymer structures for electron-tunneling based devices
Source: Sci Rep. 2020 Jul 9;10:11373. doi: 10.1038/s41598-020-68288-5 (PMC7347914; doi:10.1038/s41598-020-68288-5)
Supplement: Supplementary file 1 — Supplementary file1 (PDF 424 kb) [file 41598_2020_68288_MOESM1_ESM.pdf]

3D-Printed Graphene/Polymer Structures for Electron-Tunneling Based Devices

Deisy C. Carvalho Fernandes, Dylan Lynch, and Vikas Berry*

Department of Chemical Engineering, University of Illinois at Chicago,

810 S Clinton, Chicago, IL, 60607.

*vikasb@uic.edu

SUPPLEMENTARY INFORMATION

1. Pos (G_0)

Values for Pos(G_0) position:

G-band at Γ = 1580[1,2],1581[3], 1582[4], 1583[5], 1,584[6,7], 1585[8], 1587[9]. The average value used is 1583.67

2. I(D)/I(G) Ratio

	1	2	3	4	5	Average	St. Dev.
I(D)/I(G)	0.559642	0.554556	0.484976	0.483402	0.476558	0.511827	0.033875
L_a (nm)	34.35165	34.66668	39.64038	39.76942	40.34061	37.75375	2.429623

3. Thermal barrier Analyzes

The Arrhenius equation model was used to calculate the thermal barrier, using the model of $\frac{1}{V} \propto \exp\left(\frac{E_a}{K_B T}\right)$, the voltage applied is expressed as V, T is the absolute temperature, K_B is the Boltzmann constant, E_a is thermal activation, and I is the current (A).

$$\frac{1}{V} \propto \exp\left(\frac{E_a}{K_B T}\right)$$

Linearizing the equation

$$\ln\left(\frac{1}{V}\right) \propto \left(\frac{E_a}{K_B}\right)\left(\frac{1}{T}\right)$$

From this data it was found that the E_a= 0.15meV

4. Electron tunneling Equation fit parameters

The thermal expansion equation can be translated into the tunneling distance between the graphene and PLA 3D printed structure by:

$$\frac{a}{a_0} = (1 + \alpha * T) \qquad \text{Equation 1}$$

where a and a_0 is the tunneling distance at any point and at zero temperature, α is the thermal expansion coefficient, and T is the temperature. The Fowler Nordhiem electron tunneling governs the values shown in figure 4 by : $I = I_0 + t \exp\left(-\left(\frac{2(2m\emptyset)^{\frac{1}{2}}}{\bar{h}}\right) a_0 * (1 + \alpha * T)\right)$, where t is the tunneling proportionality constant, m is the mass of electron and ϕ tunneling barrier. The results of data used for 2-probe setup was analyzing using the Origin software to fit the data as y = c1+c2*exp(-2.24*(c3*(1+c4x)), where y is the current and x is the Temperature. The results found are:

Equation	C1+C2*exp(-2.24*(C3*(1+C4x))
Plot	B
C1 (I_0)	8.82462E-5
C2 (t)	-7.48927E-5
C3 (a_0)	0.77701 ± 0.07518 ≈0.78
C4 (α)	7.4E-4
Reduced Chi-Sqr	1.08E-15

R-Square(COD)	0.99907
Adj. R-Square	0.99814

Using c3 it was possible to calculate the tunneling distance, to be 0.89nm. Similar analyzes were done with the strain results, where a plot was done with the current and strain percentage, and the Fowler Nordhiem electron tunneling governs the values shown in figure 4 by: $I = I_0 + t\exp\left(-\left(\frac{2(2m\emptyset)^{\frac{1}{2}}}{\hbar}\right)a_0 \times \left(1+\frac{\varepsilon}{100}\right)\right)$, (equation with C1=0). Origin fitting was done using 1/y = C1 + C2/(1+ C3*exp(-2.24 * C4 * (x))). The results found was:

Equation	C1 + C2/(1+ C3*exp(-2.24 * C4 * (x)))
Plot	B
C1 (R_C) Series Leak resistance	0
C2 (I_0)	0.04956
C3(t)	10.59984
C4(a_0)	0.94574
Reduced Chi-Sqr	4.70801
R-Square(COD)	0.99396
Adj. R-Square	0.98791

Using c3 it is possible to calculate the tunneling distance of 0.95nm which aggress with the previous experiment if we use the thermal expansion equation.

5. Fowler-Nordheim Fit

Fit for the F-N type equation for the data in figure 1

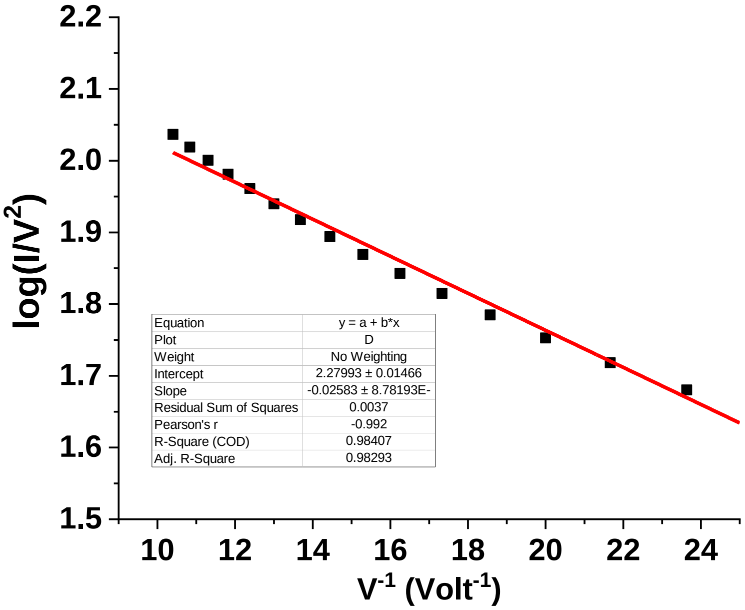

6. Strain Calculation

The strain was calculating using

$Strain = \frac{l - l_0}{l_0}$, where l us the distance of the device stretched (after strain is applied)

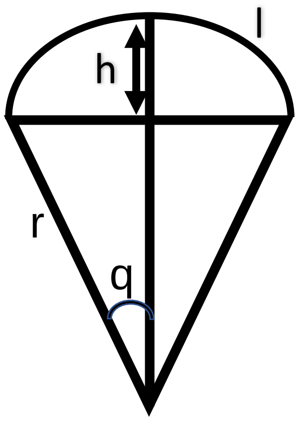

$$\rho^2 = \frac{{l_0}^2}{4} + (\rho - h)^2$$

$$\rho = \frac{{l_0}^2}{8h} + \frac{h}{2}$$

$$l = 2 * \theta * \left(\frac{{l_0}^2}{8h} + \frac{h}{2}\right)$$

$$\sin\theta = \frac{{l_0}/{2}}{\left(\frac{{l_0}^2}{8h} + \frac{h}{2}\right)}$$

$$\theta = \sin^{-1}\left(\frac{{l_0}/{2}}{\left(\frac{{l_0}^2}{8h} + \frac{h}{2}\right)}\right)$$

$$\varepsilon = \frac{\left(2 * \sin^{-1}\left(\frac{{l_0}/{2}}{\left(\frac{{l_0}^2}{8h} + \frac{h}{2}\right)}\right)\right) * \left(\frac{{l_0}^2}{8h} + \frac{h}{2}\right) - l_0}{l_0} * 100$$

7. Raman Spectra G Peak Intensity

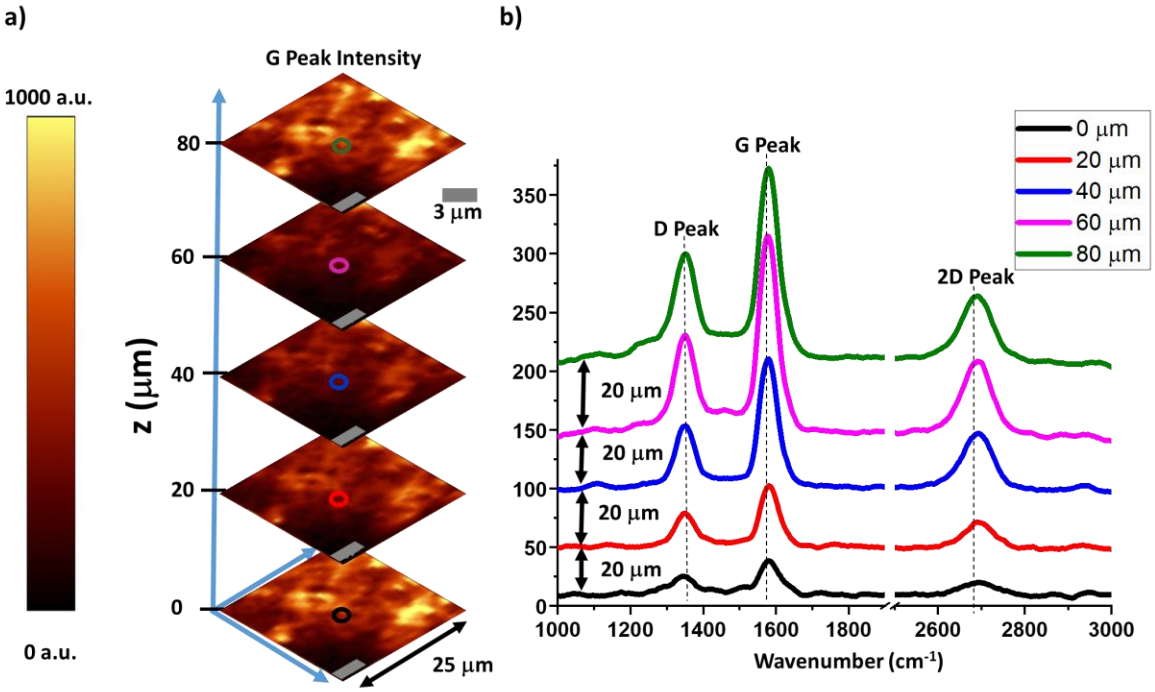

Figure S1 - a) Raman intensity mapping of graphene G peak at different depths of the mixed structure with each consecutively scan 20 μm apart showing the consistency of the graphene present throughout the structure **b)** Raman spectra of the stacked structure showing D, G and 2D peaks at different depths (each scan is 20 μm apart) of the PLA/Graphene 3D printed structure that corresponds to the mapping showing in Figure 2-(a), showing that the graphene sheets are preserved the same throughout the device.

Bibliography

1. Li, X. *et al.* Large-area synthesis of high-quality and uniform graphene films on copper foils. *Science (80-.).* (2009). doi:10.1126/science.1171245

2. Ferrari, A. C. *et al.* Raman spectrum of graphene and graphene layers. *Phys. Rev. Lett.* (2006). doi:10.1103/PhysRevLett.97.187401

3. Ouyang, Y. & Chen, L. Surface-enhanced Raman scattering studies of few-layer graphene on silver substrate with 514nm excitation. *J. Mol. Struct.* **992**, 48–51 (2011).

4. Fu, X., Bei, F., Wang, X., O'Brien, S. & Lombardi, J. R. Excitation profile of surface-enhanced Raman scattering in graphene–metal nanoparticle based derivatives. *Nanoscale* **2**, 1461 (2010).

5. Di, C. *et al.* Patterned Graphene as Source/Drain Electrodes for Bottom-Contact Organic Field-Effect Transistors. *Adv. Mater.* **20**, 3289–3293 (2008).

6. Das, A. *et al.* Monitoring dopants by Raman scattering in an electrochemically top-gated graphene transistor. *Nat. Nanotechnol.* (2008). doi:10.1038/nnano.2008.67

7. Lee, I. *et al.* Poly-4-vinylphenol and poly(melamine-co-formaldehyde)-based graphene passivation method for flexible, wearable and transparent electronics. *Nanoscale* **6**, 3830 (2014).

8. Lucchese, M. M. *et al.* Quantifying ion-induced defects and Raman relaxation length in graphene. *Carbon N. Y.* **48**, 1592–1597 (2010).

9. Wang, H., Wang, Y., Cao, X., Feng, M. & Lan, G. Vibrational properties of graphene and graphene layers. *J. Raman Spectrosc.* **40**, 1791–1796 (2009).
